# Tuning the Toxicity of Reactive Oxygen Species into Advanced Tumor Therapy

**DOI:** 10.1186/s11671-021-03599-8

**Published:** 2021-09-13

**Authors:** An Xie, He Li, Yumei Hao, Yujia Zhang

**Affiliations:** 1 Department of Pharmaceutics, College of Pharmacy, Anhui University of Chinese Medicine, Hefei, 230000 Anhui China; 2 Institute of Pharmaceutics, Anhui Academy of Chinese Medicine, Hefei, 230000 Anhui China; 3 State Key Laboratory of Bioactive Substance and Function of Natural Medicines and Beijing Key Laboratory of Drug Delivery Technology and Novel Formulation, Institute of Materia Medica, Chinese Academy of Medical Sciences and Peking Union Medical College, Beijing, 100050 China

**Keywords:** Reactive oxygen species, Photodynamic therapy, Chemodynamic therapy, Tumor therapy

## Abstract

The biological functions and toxic effects of reactive oxygen species (ROS) are generally entangled. A large amount of ROS may cause oxidative damage to cell biomolecules, leading to cell death. Tumor treatment can be carried out by using the toxicity of ROS, and various nanosystems related to ROS have been designed. In fact, the level of active oxygen in the biological microenvironment can be regulated in advanced therapeutics via designed nanoscale engineering, which can open up a new direction of treatment with specific simplicity. In this progress report, the authors first introduced how ROS causes cell death. Then, recent studies on converting the inherent toxicity from ROS into advanced treatment tools are highlighted.

## Introduction

Reactive oxygen species (ROS) are chemically active oxygen-containing atoms or groups, including singlet oxygen (^1^O_2_), superoxide anion (O_2_-), hydroxyl radical (^·^OH) and hydrogen peroxide (H_2_O_2_) [1–4]. Mitochondria is the main place for the generation of ROS in the cell, mainly through the electron transport chain, such as O_2_-·, ^·^OH and ^1^O_2_ are all by-products of aerobic metabolism [5]. In most cells, more than 90% of the oxygen is consumed in the mitochondria, and 2% of the oxygen is converted into oxygen free radicals in the inner mitochondrial membrane and matrix [5, 6]. ROS has vital function in maintaining tissue homeostasis,regulating signal transduction and differentiation, and promoting cell damage and death. The level of ROS is controlled by the cellular antioxidant defense system [7–10].

**Fig. 1:**
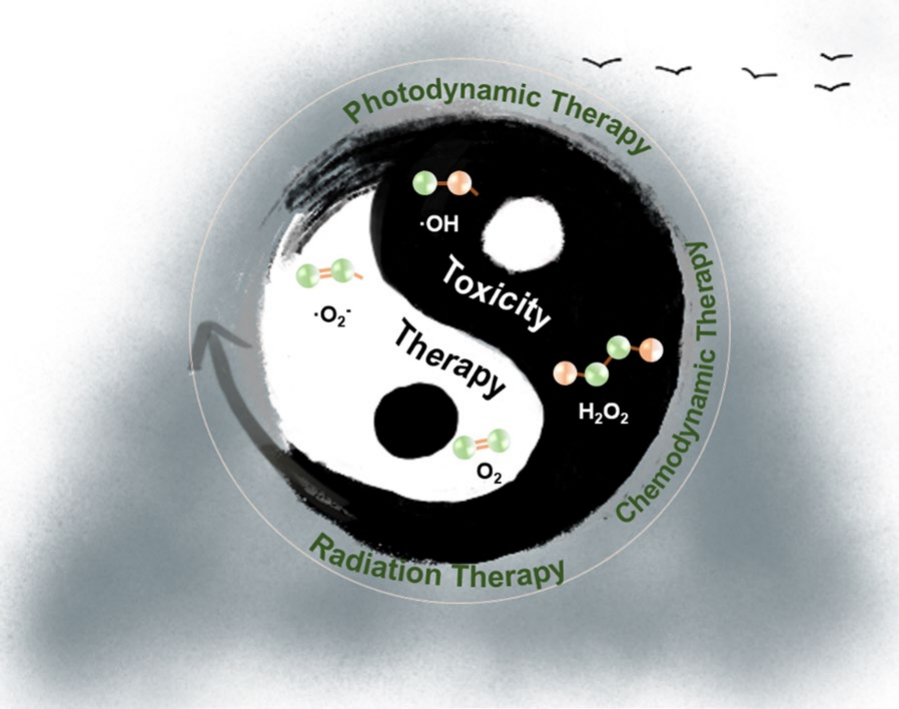
Schematic representation of the the potential of transforming toxicity to therapy

ROS is the main molecule produced during oxidative stress in the body, and has been considered an important factor in tumor occurrence, development and recurrence [11]. ROS include groups with unpaired electrons containing oxygen atoms and excessive ROS can damage biological macromolecules such as DNA and proteins in tissues. The increase of ROS will increase the mutation rate and promote the transformation of normal cells into tumor cells. ROS can also promote the stability of important signal molecules that drive tumorigenesis and progression. That is to say, ROS are not only a factor of tumor production, but also a factor of tumor deterioration. However, the increase of ROS in tumor cells can cause cell death, which can inhibit the further growth of tumor. Taken together, ROS can play multifaceted roles in tumor [12, 13]. Both detrimental and beneficial effects were found for ROS-mediated mechanisms with varying success [14–16]. The past decades have witnessed a tremendous growth of ROS-related nanotheranostics which are emerging as an important direction to future nanomedicine implicating a close crosstalk between multidisciplinary fields [17, 18]. To this end, it is important to decipher the logic between ROS generation and elimination to revolutionize the design considerations. In this progress report, we first provide the biological effects of ROS. Then, we discuss anti-tumor strategies based on ROS. Among them, we highlight recent studies for using ROS toxicity as a highly effective therapeutic tool for tumors (Fig. 1).

## Fight Fire with Fire

ROS has been reported to be associated with cancer development and cancer cell death. Once the toxicity of ROS can be controlled well, applying ROS-related nanomedicines seems to be a promising approach to tumor therapeutic applications [7, 19, 20]. First, a large number of studies of the mechanism toxicity of ROS have provided a strong foundation for the development of methods for the transformation of toxicity to therapeutic effects [5, 6]. Moreover, from a practical perspective, many scientists have already demonstrated the feasibility of modification of nanomedicines to alter their physicochemical properties, enabling the precise control of ROS level at specific sites. Therefore, ROS-related nanomedicines have the immense potential to be an independent therapeutic tool. Indeed, some proof-of-concept studies have already specifically addressed this potential.

ROS promoted tumor development by inducing DNA mutation and genomic instability or as a signal molecule, accelerating tumor cell proliferation, survival and metastasis. However, excessive ROS enhance cellular oxidative stress, resulting in DNA, protein or lipid damage, and lead to cell apoptosis or necrosis [21, 22]. Therefore, boosting ROS in tumor cells through nanomedicines has been applied to the treatment of clinical cancer. In the following sections, we will survey the approaches enabling to increasing the intracellular ROS level, including photodynamic therapy (PDT), chemodynamic therapy (CDT) and radiation therapy (RT) in cancer therapy, thus facilitating the future development of new strategies to overcome the limitations of current ROS-based cancer therapies.

### Photodynamic Therapy

**Fig. 2:**
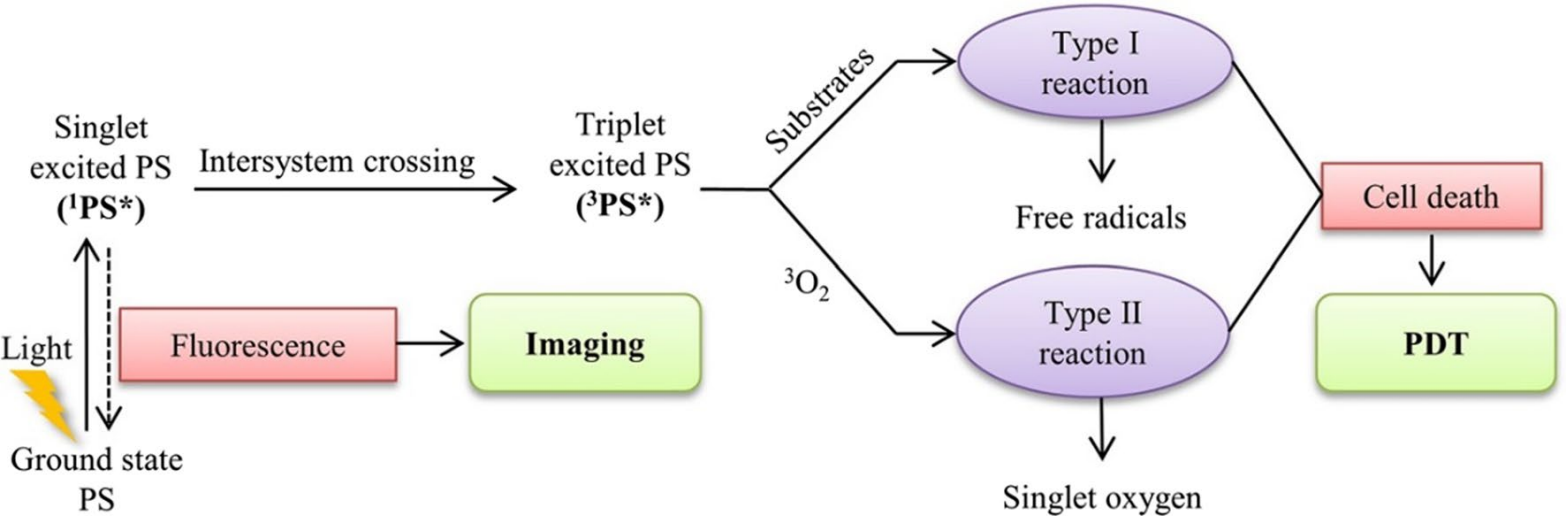
Schematic drawing of a typical photodynamic reaction [23]

In a typical PDT system, photosensitizers (PSs), light and oxygen are the three essential components of PDT. The PS is transformed from its ground state to its triplet excited state through a short-lived singlet state as a result of excitation with light of a specific wavelength, and lead to generate excess cytotoxic ROS, then the ROS ultimately induces the regression of targeted lesions [23–25]. Figure 2 shows the mechanisms of PDT: In type I mechanisms, the PS reacts directly with an organic molecule in a cellular microenvironment, acquiring a hydrogen atom or electron to form a radical, leading to the production of ROS and macromolecule degradation, which is cytotoxic to the cell [23]. In type II mechanisms, the PS in triplet state can either decay radiation lessly to the ground state or transfer its energy to molecular oxygen, which is unique in being a triplet in its ground state, leading to the formation of cytotoxic ROS, such as singlet oxygen (^1^O_2_). Unfortunately, owing to weak light absorption in the optical transparent window of biological tissues, most available PSs such as photofrin exhibit low^1^O_2_ quantum yields when excited by the light within the phototherapeutic window [26]. Furthermore, the application of PDT has been limited by the poor bioavailability of the PSs, and low levels of oxygen in tumors can further decrease ^1^O_2_ production [27, 28]. Therefore, the design and exploitation of suitable PSs plays a vital role in promoting the development of PDT. Nanomaterials as a promising technique for PDT that can overcome most of the limitations of traditional PSs. This section combs the recent examples of that increase the level of intracellular ROS to enhance PDT, including various types of nanomaterials.

**Fig. 3:**
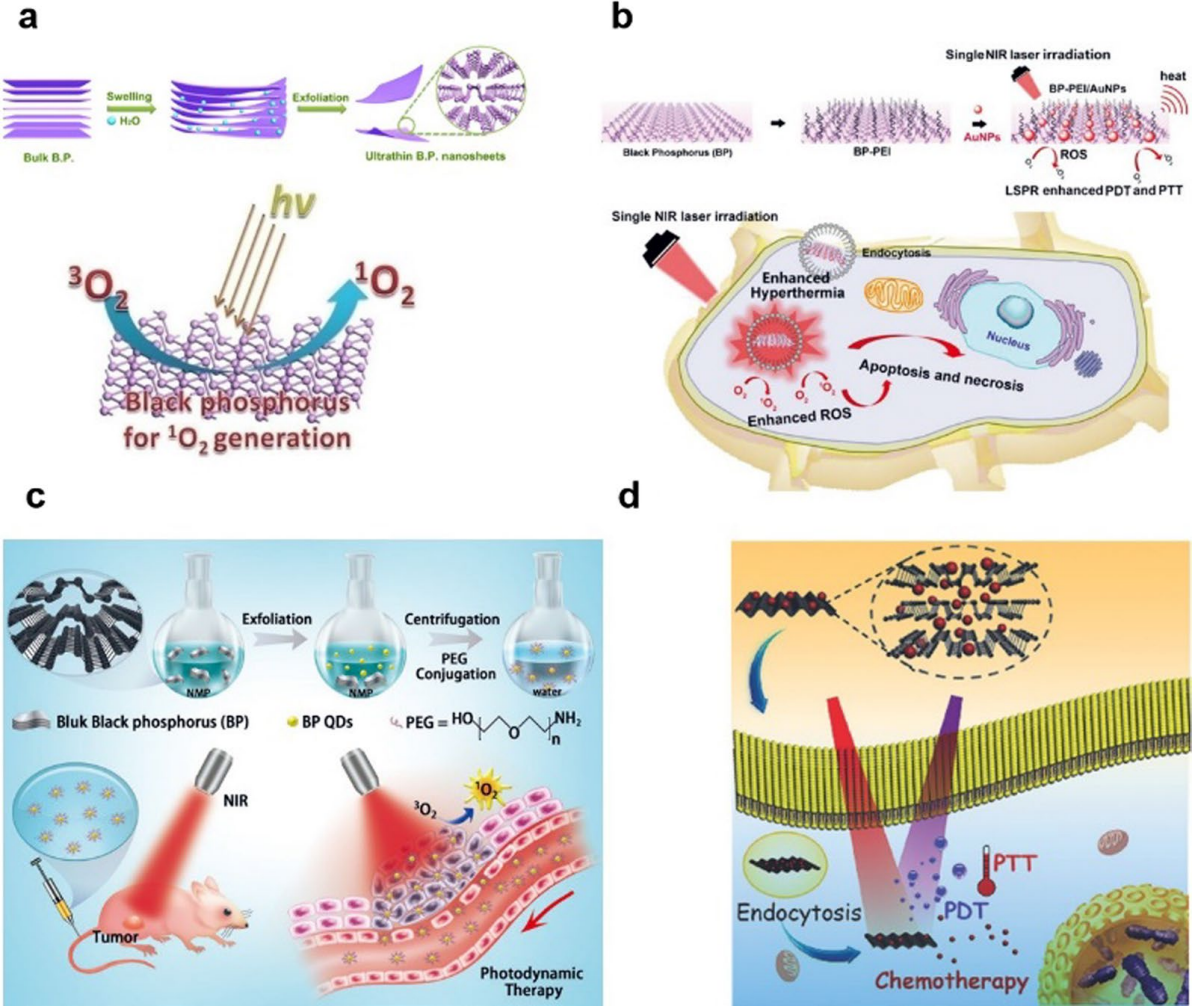
**a** Schematic diagram for the water exfoliation of bulk B.P. into ultrathin nanosheets. **b** The preparation and schematic view of the biofunction of BP-PEI/AuNPs. In cancerous cells, the enhanced PTT/PDT by localized surface plasmon resonance (LSPR) could simultaneously enhance hyperthermia and singlet oxygen for cancer phototherapy. **c** Schematic diagram of the synthesis of BPQDs and their potential application in PDT. **d** Abridged general view of BP-based drug delivery system for synergistic photodynamic/photothermal/chemotherapy of cancer [29–32]

Black phosphorous nanosheets (BP NSs) with unique energy-band structures generate ^1^O_2_ under 660 nm near-infrared (NIR) light irradiation; therefore, they can be developed as highly effective PSs for PDT. Furthermore, studies have shown that the BP NSs can be degraded and have good biosafety performance [29] (Fig. 3a). Zhang et al. designed the BP-PEI/AuNPs hybrid nanosheet, which hybridized BP NSs employed as two-dimension (2D) inorganic PSs with gold nanoparticles (AuNPs) through polyetherimide (PEI). The significantly improved PDT effects of BP-PEI/AuNPs nanosheet resulted in the effective inhibition of the tumor growth both in vitro and in vivo (Fig. 3b) [30]. Yang et al. successfully developed, BP quantum dots (BPQDs) and investigated their potential to serve as PDT agents. The BPQDs showed good stability in physiological medium and no observable toxicity after PEG conjugation. In addition, the BPQDs could effectively generate ^1^O_2_ under light irradiation. Both in vitro and in vivo studies demonstrated that the BPQDs exhibited excellent antitumor efficiency through the PDT (Fig. 3c) [31]. Guo et al. reported a new class of multimodal therapeutic system based on BP NSs. Using DOX as a modal drug, BP possessed extremely higher drug loading capacity for DOX. Under near-infrared light, BP NSs can effectively generate ^1^O_2_ under NIR light irradiation. The intrinsic properties of BP NSs allowed them to simultaneously serve as both effcient PDT and PTT agents (Fig. 3d) [32].

**Fig. 4:**
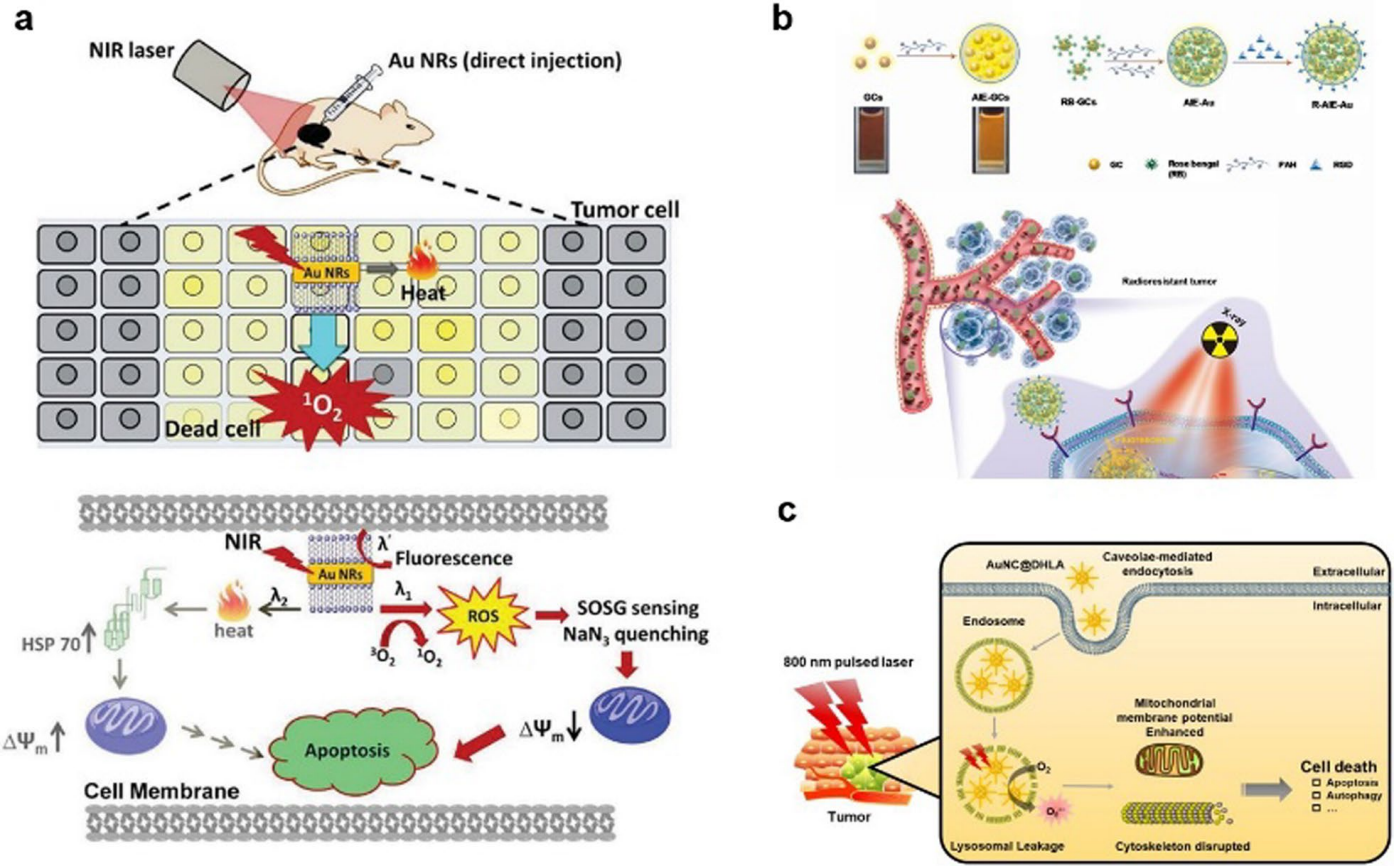
**a** Schematic working mechanisms of photothermal and photodynamic therapy effects exerted by Au NRs at low light doses. **b** Schematic diagrams of the working model of R-AIE-Au for fluorescence and CT imaging-guided X-ray-induced enhanced RT and PDT. **c** Schematic illustration of the cancer therapy mechanism of AuNC@DHLA PDT. AuNC@DHLA can be internalized via caveolae-mediated endocytosis and accumulate in lysosomes, where the generation of ROS leads to LMP. Subsequent altered MMP, mitochondrial morphology, and cytoskeleton destruction finally lead to cell death. The in vivo PDT was achieved with NIR fs laser irradiation [34–36]

Gold-based nanoparticles, have also been extensively studied for the application in PDT [33]. Hwang et al. have presented the first literature example of nanomaterials-mediated PDT and demonstrated that upon NIR light irradiation, Au NRs can mediate PDT effects to completely destruct tumor in mice in the absence of additional organic photosensitizers (Fig. 4a) [34]. Chen et al. designed aggregation‐induced emission gold clustoluminogens (AIE‐Au) to achieve efficient low‐dose X‐ray‐induced PDT (X‐PDT) with negligible side effects. X‐ray‐induced luminescence excited the conjugated photosensitizers, resulting in a PDT effect. The in vitro and in vivo experiments demonstrated that AIE‐Au effectively triggered the generation of ^1^O_2_ with an order‐of‐magnitude reduction in the X‐ray dose, enabling highly effective cancer treatment (Fig. 4b) [35]. Jiang et al. developed the dihydrolipoic acid coated AuNCs (AuNC@DHLA) as PSs for efficient in vivo PDT. In contrast to the ^1^O_2_ (type II) mechanism of most conventional PSs, the photochemical mechanism of AuNC@DHLA involved the type I process. With AuNC@DHLA as the PSs, highly efficient in vivo PDT has been achieved (Fig. 4c) [36].

Although PDT has been applied clinically in recent years, it has not yet become a first-line treatment. It largely depends on the complex photosensitivity of PDT, which requires fine coordination between light, PS and oxygen (O_2_), which greatly limits the efficacy of PDT. In recent years, in order to improve the efficiency of PDT-mediated ROS generation, many methods have been developed, such as the use of new nanomaterials as light sensors to increase the depth of light penetration, and the use of nano-drug complexes as O_2_ supply systems to solve tumor tissues. However, the relationship between the retention time and spatial distribution of the oxygen provided by the nanosystem and the effectiveness of the nanosystem to enhance the anti-tumor effect needs further study.

### Chemodynamic Therapy

Chemodynamic therapy (CDT) is an emerging cancer treatment method that uses the Fenton/Fenton-like reaction between metals and peroxides to generate highly reactive hydroxyl radicals (^·^OH) to achieve efficient tumor cell killing [37–47]. At present, the main method to achieve CDT is to deliver Fenton-active transition metal ions, thereby triggering the conversion of intracellular H_2_O_2_ to ^·^OH that to induce oxidative stress and subsequent cancer cell death through the oxidation of various biomolecules such as DNA and proteins [13, 16, 46–53]. The Fenton reaction has been written as Eqs. (1) and (2) [46].

1 $${\text{Fe}}^{{{2} + }} + {\text{H}}_{{2}} {\text{O}}_{{2}} \to {\text{Fe}}^{{{3} + }} +^{\cdot} {\text{OH}} + {\text{OH}}^{ - }$$

2 $${\text{Fe}}^{{{3} + }} + {\text{H}}_{{2}} {\text{O}}_{{2}} \to {\text{Fe}}^{{{2} + }} +^{\cdot} {\text{HO}}_{{2}} + {\text{H}}^{ + }$$

The Fenton reaction is a process in which H_2_O_2_ reacts with ferrous ions to produce ^·^OH with strong oxidizing properties. Since the content of H_2_O_2_ in tumors is significantly higher than that in normal tissues, the generation of ^·^OH based on Fenton reaction is a preferred solution for the use of ROS to achieve selective tumor therapy; the effective and specific transport of ferrous ions to the tumor sites has become a research hotspot. Benefiting from the weak acid microenvironment characteristics of tumor, acid-sensitive iron-based nanomaterials can achieve selective release of ferrous ions at tumor sites, which is expected to achieve efficient and specific treatment of tumors.

**Fig. 5:**
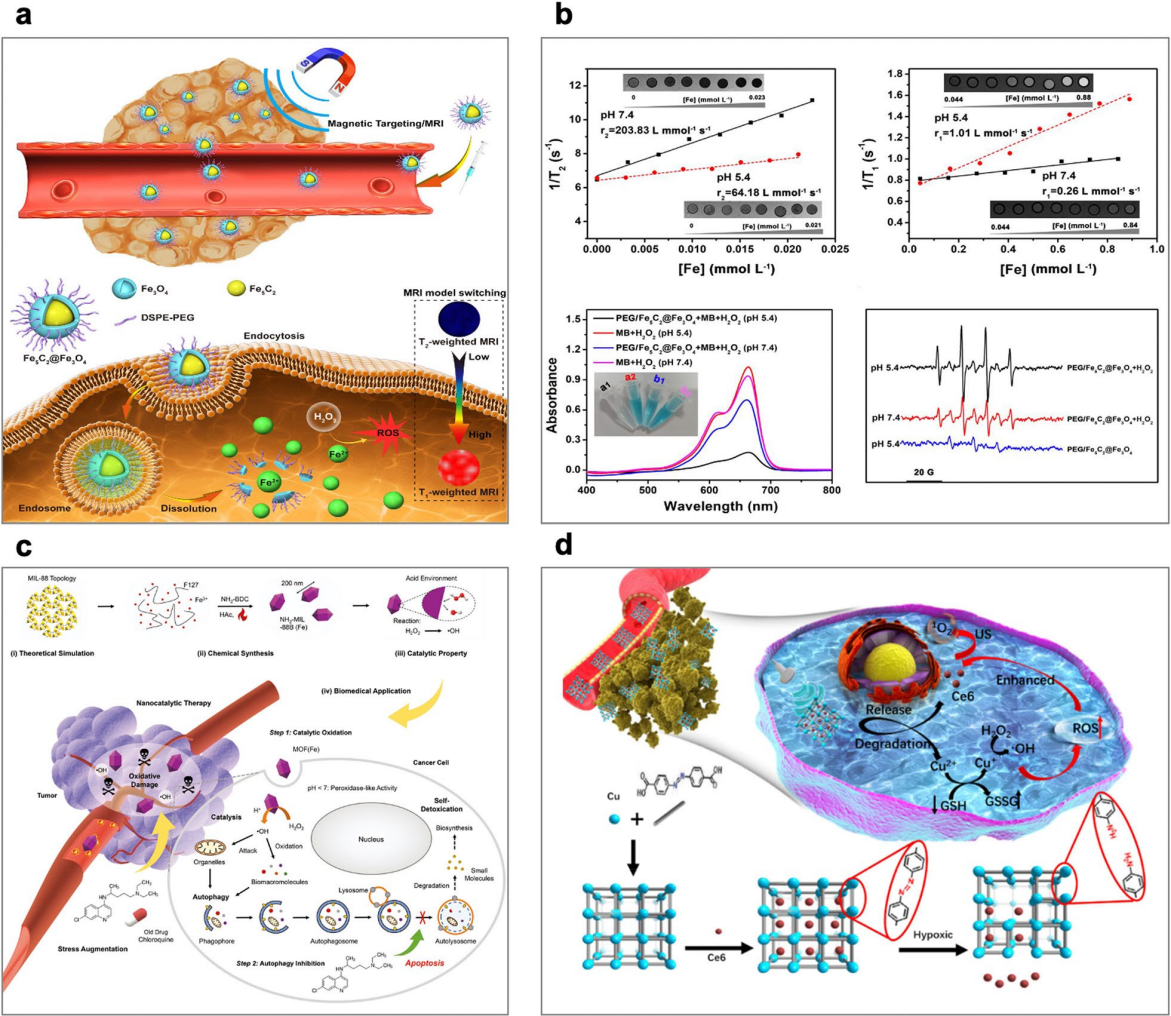
**a** A diagram of Fe_5_C_2_@Fe_3_O_4_ NPs for pH-responsive Fe^2+^ releasing, ROS generation and T2/T1 signal conversion. **b** pH-dependent MRI model switching of PEG/Fe_5_C_2_@Fe_3_O_4_ nanoparticles. **c** Schematic illustration for the underlying material chemistry and therapeutic concept: MOF (Fe) catalyzes Fenton-like reactions in cancer cells to convert the intrinsic nontoxic H_2_O_2_ into highly oxidative ^·^OH, which attacks and inactivates ambient protein and organelles and leads to their aberrant accumulation. **d** Schematic of the synthetic procedure and hypoxia-responsive copper metal–organic frameworks nanosystem for improved cancer therapy [54, 55, 65]

To this end, Hou et al. developed a switchable MRI-guided cancer therapeutic agent based on ROS generation by Fe_5_C_2_@Fe_3_O_4_ NPs. The Fe_5_C_2_@Fe_3_O_4_ NPs are pH-sensitive, releasing ferrous ions in acidic tumor environments, and the discharged Fe^2+^ ions disproportionate the H_2_O_2_ that is overproduced at tumor sites to generate ^·^OH for effective tumor therapy (Fig. 5a) [54]. Moreover, they have high magnetic properties, which are beneficial as they allow visualization of tumor aggregation through magnetic targeting and T2-weighted MRI. The effective tumor orientation and ROS generation were confirmed through both in vitro and in vivo experiments, which showed excellent therapeutic efficacy with low toxicity. In addition, the dissolution of Fe_5_C_2_@Fe_3_O_4_ NPs in the low-pH region reduces the T2 signal on MRI, and the release of ferrous ions raises the T1 signal, providing an MRI-supervised tumor therapy. These Fe_5_C_2_@Fe_3_O_4_ NPs are the pioneering paradigm of the application of iron carbide for tumor regression based on the selective catalysis of the Fenton reaction without the need for external energy input, providing a visible strategy for efficient and specific tumor therapy (Fig. 5b). In another example, Shi et al. explored an iron-containing metal–organic framework [MOF(Fe)] nanocatalyst as a peroxidase mimic is used to catalyze the generation of highly oxidizing ^·^OH radicals specifically within cancer cells, while chloroquine is applied to deacidify lysosomes and inhibit autophagy, cutting off the self-protection pathway under severe oxidative stress(Fig. 5c). Cancer cells fail to extract their components to detoxicate and strengthen themselves, finally succumbing to the ROS-induced oxidative damage during nanocatalytic therapy. Both in vitro and in vivo results demonstrated that such a combinational therapeutic approach results in remarkable antineoplastic effects, which may be hopefully to the design of treatment regimens in the future [55].

Apart from the production of ROS mediated by iron ions or iron based NPs, other metal ions, such as Mn^2+^, Cu^2+^, Ag^+^ and Pt^2+^, as well as their corresponding NPs, also show a Fenton-like activity [56–64]. Zhang et al. reported a copper metal–organic framework nanoparticles (Cu-MOF NPs) that copper clusters bridged by organic ligands loaded with sonosensitizers chlorin e6 (Ce6), which show good tumor accumulation, on-demand release numerous Cu^2+^ and Ce6 in responding to hypoxia TME, achieving glutathione (GSH)-depleted chemodynamic/sonodynamic therapy (CDT/SDT) (Fig. 5d) [65]. In detail, the large size Cu-MOF NPs were effectively accumulated in the tumor via enhanced permeability and retention effect (EPR), and the hypoxia TME triggered the degradation of Cu-MOF NPs to release the Cu^2+^ and Ce6 and deep tumor penetration. The redox between free Cu^2+^ and intracellular high-level GSH, resulting in GSH depletion and reducing Cu^2+^ to Cu^+^. The Cu^+^ catalytic Fenton-like reaction shows a high catalytic activity and specificity in weakly acidic TME, which exhibited cytotoxicity to cancer cells. The GSH depletion and Ce6-mediated SDT further enhanced therapy efficiency. In vivo results showed the Cu-MOF NPs selectively and effectively killed cancer with high specificity and minimal invasivenes.

In recent years, CDT has made rapid progress in the field of tumor therapy, but there are still some challenges in the process of clinical transformation. For example, a series of challenges such as the mass repeatable synthesis of nanomaterials, the biosafety of nanomaterials, the evaluation criteria for the therapeutic effects of nanomaterials and the deeper biological principles, still require the concerted efforts of researchers from multiple disciplines to solve.

### Radiation Therapy

**Fig. 6:**
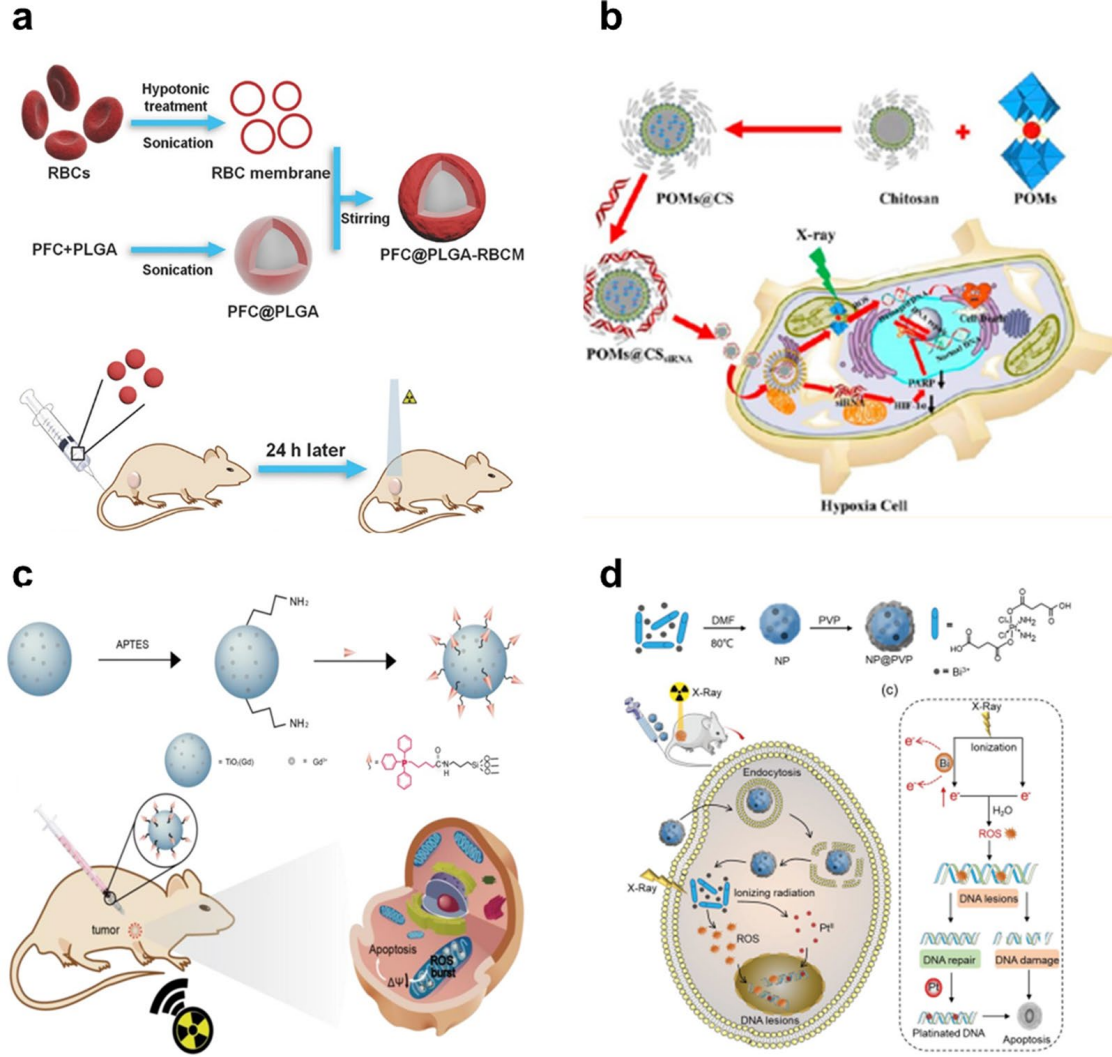
**a** Mechanism diagram of the PFC@PLGA-RBCM NPs for RT treatment. **b** Schematic representation of the GdW10@CS NP for efficient radiosensitization efficacy of RT against hypoxic tumor cells. **c** Mitochondria-targeted nanosensitizer TiO2 (Gd)-TPP NPs for radiotherapy to trigger mitochondrial ROS accumulation. **d** Schematic diagram of the synthesis of NP@PVP with bismuth and cisplatin prodrug and the mechanism of enhanced chemo-radiotherapy efficacy under X-ray irradiation

Radiation therapy (RT) is one of the most widely used methods in the treatment of cancer and plays a very important role in the treatment of cancer [66]. RT, takes advantage of high-intensity ionizing radiation to suppress tumor proliferation with no depth restriction, during which it can induce DNA double-strand damage by generating considerable cytotoxic reactive oxygen species (ROS) produced by the ionization of surrounding water [66–69]. Therefore, to enhance ionizing radiation-induced cellular damage during radiotherapy, adequate ROS generation is essential to induce DNA double strand damage by reacting with DNA and greatly suppressing reconstruction of the broken double-stranded DNA [70]. RT mainly uses ionizing radiation to irradiate tumor tissues to destroy the DNA of cancer cells by generating large amounts of cytotoxic reactive oxygen (ROS) [71]. Ionization can cause atomic and molecular bonds to break, and DNA double-strand breaks are currently believed to be the main cause of cell death. However, some types of tumors or even intratumoral areas may be less sensitive to the cancer-killing effects of RT, due to mechanisms such as hypoxia during treatment and accelerated tumor cell reproliferation, which may lead to the aggregation of tumor cells that survive RT. Liu et al. developed the PFC@PLGA-RBCM NPs, in which the PFC core can dissolve large amounts of oxygen (O_2_) and the red-blood-cell membrane (RBCM) coating would enable greatly extend blood circulation for those nanoparticles. PFC@PLGA-RBCM NPs could effectively deliver O_2_ to the tumor after intravenous administration, thus greatly relieved tumor hypoxia and significantly enhanced treatment efficacy of RT (Fig. 6a) [72]. Zhao et al. designed a GdW10@CS NPs for enhanced radiosensitization of RT in hypoxic tumors. The GdW10@CS NPs simultaneously utilizes the GdW10@CS as an external radiosensitizer to deposit radiation dosage and obliterate the intracellular GSH for more effective ROS generation. and HIF-1α siRNA as an internal stimulation method to inhibit double-stranded DNA repair to realize a radiosensitization effect of radiotherapy. the HIF-1α siRNA as an internal stimulus to inhibit double-stranded DNA repair and achieve radiosensitizer effects of RT(Fig. 6b) [73].

In order to enhance the cell damage induced by ionizing radiation in radiotherapy, it is essential to be able to generate enough ROS, which can induce DNA double-strand damage by reacting with DNA and greatly inhibit the remodeling of broken double-stranded DNA [74–76]. Recent studies have shown that increasing ROS level in tumor cells during RT can significantly improve RT efficiency and reduce radiotherapy dose, thus reducing non-selective killing of normal cells and serious systemic side effects on bystander organs. For instance, Tang et al. developed a mitochondrial targeting, Gd-doped titanium dioxide nanosensitizer called TiO_2_ (Gd)-TPP NPs for effective RT. Because the nanosensitizer has a large photoelectric cross section for X-rays, it can effectively produce ROS. The experimental results demonstrated that mitochondria-targeted nanosensitizers could significantly reduce the treatment dose and enhance the anti-tumor efficacy. This strategy may provide an effective and universal method to improve tumor radiosensitivity in future clinical cancer treatment (Fig. 6c) [77]. Zhan et al. constructed a nano-coordination platform (NP@PVP) for bismuth nitrate and cisplatin precursors, namely a radiosensitizer. Bismuth in NP@PVP can sensitize RT by increasing the production of ROS and enhancing DNA damage after X-ray irradiation in tumor cells. NP@PVP had higher sensitization enhancement ratio (SER was 2.29) and better tumor ablation ability in compared with cisplatin (SER was 1.78) (Fig. 6d) [78].

Consequently, many studies have shown that the strategy of nanomedicine mediated ROS generation to achieve RT sensitization has great anti-cancer potential in RT, and has a good clinical application prospect. With the development of tumor molecular biology, the research and understanding of nanomedicine radiotherapy sensitization should go deep into molecular biology and gene level, and then a more essential and universal explanation mechanism of radiotherapy sensitization should be proposed. Therefore, it is necessary to strengthen the research on the mechanism of radiotherapy sensitization based on nanomaterials that promote the production of reactive oxygen species. This can not only clarify the radiosensitization mechanism of nanomaterials, and provide a basis for its application in the biological field; it also helps to further understand the interaction between nanomedicine, high-energy rays and biological tissues, thereby improving the structure and performance of nanomedicine. Expanding the scope of application, discovering new application areas, reducing toxic and side effects, etc. have guiding significance.

## Conclusions and Outlook

This review aims to reveal and resolve the therapeutic effects of toxicity caused by ROS. In order to promote the shift in the role of reactive oxygen species from pathogenic factors to therapeutic factors, and facilitate successful therapeutic conversion, we should consider the principle of its toxicity and design ROS-related nanosystems.

ROS played an important role in the process of life, and high levels of ROS can cause oxidative damage to cell biomolecules, leading to cell death. We can use its toxicity to treat according to its mechanism of action to achieve the effect of “like cures like”. Therefore, ROS-based tumor treatment strategies show great promise. In recent years, there have been many studies devoted to the development of integrated ROS-regulation nanomaterials and many strategies have been developed to solve the existing problems in redox modulation therapy. This mini-review summarized the development and application of various ROS-related nanosystems for tumor treatment in recent years, involves ROS-induced toxicity treatment, and proposes some basic and key principles for the design of ROS-related nanosystems. Although the development of ROS-regulating therapy has made significant progress in recent years, the design of ROS-related nanosystems is still in its infancy, and there are still many challenges to be solved. PDT uses photosensitizers to generate ROS to kill tumor cells under light activation. However, the tumor hypoxia and limited light penetration depth limit its development. Compared with PDT, CDT is an emerging treatment strategy that uses biochemical reactions to produce ROS to kill tumor cells, which depends on neither molecular oxygen (O_2_) nor external light source, enabling chemodynamic therapy to avoid the major shortcomings of photodynamic therapy [79–87]. Despite its great therapeutic potential, CDT technology remains in its infancy. RT is the main treatment for various types of cancers clinically, and up to 50% of cancer patients receive this treatment modality. RT can effectively kill cancer cells by destroying the DNA double strand, but the self-repair mechanism of DNA in cancer cells highly limits its therapeutic effect. In addition, the insensitivity of hypoxic tumors to RT and the inevitable side effects at therapeutic doses also limit its efficacy.8 10 Meanwhile, normal tissues can also be injured like cancerous tissues because of non-selective absorption of X-rays. Thence, there are major problems caused by RT that need to be overcome with great efforts. High-efficiency radiosensitizers are important factors for improving RT efficacy, and it is very important to design new effective radiosensitizers for enhancing the absorption of X-rays, thereby achieving an effective therapeutic effect below the safe dose.

Generally, only PDT or CDT, RT treatment can not completely eliminate tumors, especially for metastatic tumors. It is possible to develop intelligent nanomedicines that can be used in synergy with multiple treatment methods, and can achieve synergistic treatment effects.As a whole, based on our growing understanding of ROS and the development of nanomaterials, undoubtedly, there are sustained discoveries of novel ROS-related nanosystems that are beneficial, andmay continuously lead to advanced therapeutics. In the future, researchers still need to continue to develop intelligent nano-reactive oxygen-related nanomaterials to selectively amplify the oxidative stress in tumor cells that can induce tumor cell death.

## Data Availability

Not applicable.

## Abbreviations

ROS: Reactive oxygen species
^1^O_2_: Singlet oxygen
O_2_-: Superoxide anion
^·^OH: Hydroxyl radical
H_2_O_2_: Hydrogen peroxide
PDT: Photodynamic therapy
CDT: Chemodynamic therapy
RT: Radiation therapy
PSs: Photosensitizers
O_2_: Oxygen
